# ﻿Karyotype and *COI* gene sequence of *Chironomusheteropilicornis* Wülker, 1996 (Diptera, Chironomidae) from the Gydan Peninsula, Russia

**DOI:** 10.3897/CompCytogen.v15i4.73135

**Published:** 2021-12-07

**Authors:** Viktor V. Bolshakov, Alexander A. Prokin, Sergey V. Artemenko

**Affiliations:** 1 Papanin Institute for Biology of Inland Waters Russian Academy of Sciences, Yaroslavl reg., Nekouz prov., Borok, 152742, Russia Papanin Institute for Biology of Inland Waters Russian Academy of Sciences Borok Russia; 2 Cherepovets State University, Lunacharski 5,Cherepovets, 162600, Vologda Oblast’, Russia Cherepovets State University Cherepovets Russia; 3 AquaBioSafe Laboratory, University of Tyumen, 625003, Tyumen, Russia University of Tyumen Tyumen Russia

**Keywords:** Chironomidae, *Chironomusheteropilicornis*, *COI*, Diptera, DNA-barcode, Gydan Peninsula, karyotype

## Abstract

The karyotype features and gene *COI* sequence of *Chironomusheteropilicornis* Wülker, 1996 from the Gydan Peninsula are presented for the first time. Nine banding sequences were determined, eight of them hpiA2, hpiB1, hpiC1, hpiC2, hpiD1, hpiE1, hpiF3 and hpiG1 were previously known from European, Georgian (South Caucasus) and Siberian populations. One new banding sequence for *Ch.heteropilicornis*, hpiB2, was found. The hpiA2 banding sequence was found in all individuals, and this is its second finding after the Georgian population (Karmokov 2019). The hpiF3 banding sequence was found only in the homozygous state. Additional B-chromosomes are absent. The genetic distances (K2P) between *Ch.heteropilicornis COI* gene sequence from Gydan Peninsula and Norway are 1.1­–1.3%, and Georgia – 1.8%, much lower than the commonly accepted threshold of 3% for species of genus *Chironomus* Meigen, 1803. The phylogenetic tree for *COI* gene sequences estimated by Bayesian inference showed geographically determined clusters of Norway and Gydan and a separate lineage of the Georgian population of *Ch.heteropilicornis*. The analysis of karyotype and *COI* gene sequences shows that the population of *Ch.heteropilicornis* from the Gydan Peninsula has an intermediate position within the *Ch.pilicornis* group between Georgian, Yakutian and Norwegian populations. The position of *Ch.pilicornis* Fabricius, 1787 from Canada and Greenland on the phylogenetic tree is discussed.

## ﻿Introduction

The water bodies of the Gydan Peninsula remain poorly studied. In 2012 during the investigation of the zonal distribution of macrozoobenthos in lakes of the Tyumen Oblast’, in the Tundra zone, larvae of *Chironomus* Meigen, 1803 were not recorded (Aleshina and Uslamin 2012). Later, the single species *Chironomusheterodentatus* Konstantinov, 1956 identified by larval morphology, was recorded from two unnamed inundated lakes on the Gydan Peninsula (Stolbov et al. 2017).

*Chironomusheteropilicornis* Wülker, 1996 belongs to *Chironomuspilicornis*-group, which includes one more species *Ch.pilicornis* Fabricius, 1787. In Russia larvae with unknown karyotype were found in a few populations of Sakha Republic (Yakutia): channel in the vicinity of the Yakutsk city; Bakyl pond in Khoro village, Verkhnevilyuyskiy District; Erien-Kuta lake in Antonovka village; unnamed pond for irrigation in Nyurba village; unnamed lake in Antonovka village, Nyurbinskiy District; Irelyakh River near Mirnyy city, Mirninskiy District. These larvae were initially named *Chironomus* sp. *Ya2* (Kiknadze et al. 1996), later identified as *Ch.heteropilicornis* (Kiknadze and Istomina 2000). One population is known from an unnamed lake in the Republic of Georgia (South Caucasus), Kvemo Kartli reg., Tsalka District (Karmokov 2019). This species was also recorded from Sweden, Finland (Wülker 1996), and North Germany (Kiknadze and Istomina 2011; Kiknadze et al. 2016).

At present, 16 banding sequences are known for the banding sequences pool of *Ch.heteropilicornis*: 15 of them are described by Kiknadze et al. (2016), and one additional banding sequence hpiA2 described from Georgia (Karmokov 2019).

The *COI* gene sequences of *Ch.heteropilicornis* from Norway and Georgia are present in genetic information databases, GenBank and Barcode of Life Data Systems (BOLD). In addition, COI sequences of *Ch.pilicornis* from Canada, Greenland, and Sweden were also present in aforementioned databases.

The present research aims at describing the karyotype and *COI* gene features of the *Ch.heteropilicornis* from the Gydan peninsula (Russia) in a comparison with known populations.

## ﻿Material and methods

**Figure 1.**
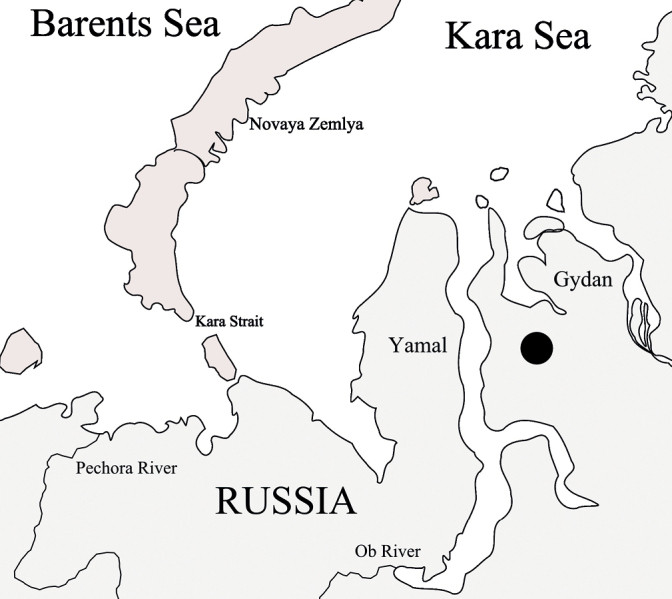
Collection site of *Ch.heteropilicornis* in Gydan Peninsula, Russia. The collection site is marked by a black circle.

Four IV instar larvae were collected from a small bay overgrown with sedge (*Carex* sp.) of an unnamed lake in Gydan Peninsula, Tazovskiy District, Yamalo-Nenets Autonomous Region (Fig. 1): 70°24'51.54"N, 76°06'42.08"E (70.414317, 76.111689) in August 4, 2018. Depth – 0.8 m, bottom – silt, detritus; water temperature – 10.5 °C, mineralization – 0.06 ppm. The total abundance of *Chironomus* spp. specimens in this habitat was estimated at 700 ind./m^2^ (67% of the total number of benthic animals) and total biomass was 6.6 g/m^2^ (38%). All larvae were used for karyotype analysis by the ethanol-orcein technique (Dyomin 1989). A Micromed-6C (LOMO, St. Petersburg) light microscope equipped with standard (kit) oil objective x100, and camera ToupCam5.1 (China) were used for microscopy analysis.

**Figure 2.**
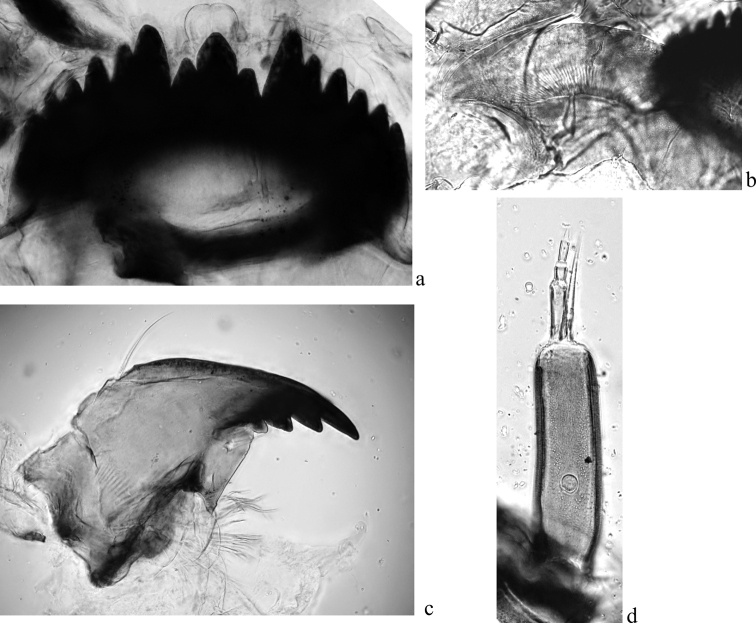
Larva morphology of *Ch.heteropilicornis* from the Gydan peninsula, Russia a mentum b ventromental plate c mandible d antenna.

The head capsule of one larva was mounted on a slide in the Fora-Berlese solution (fig. 2), the morphological terminology proposed by Sæther (1980) was used.

The larvae were determined by karyology. To identify chromosome banding sequences in arms A, E and F the cytophotomaps of Wülker (1996), Kiknadze et al. (1996, 2016), Karmokov (2019) were used, the mapping performed in the system of Keyl (1962), and for arms C and D cytophotomaps of Wülker (1996), Kiknadze et al. (1996, 2016) were used in the system of Dévai et al. (1989).

One larva which was studied karyologically was taken for the total DNA extraction using a «M-sorb-OOM» (Sintol, Moscow) kit with magnet particles according to the manufacturer’s protocol. For amplification of *COI* gene (cytochrome oxidase subunit I) we used primers LCO1490 (5’-GGTCAACAAATCATAAAGATATTGG-3’) and HCO2198 (5’-TAAACTTCAGGGTGACCAAAAAATCA -3’) (Evrogen, Moscow) (Folmer et al. 1994). The amplification reaction was carried out in 25 μl reaction mixture (1x buffer, 1.5 μМ MgCl2, 0.5 mM of each primer, 0.2 μМ dNTP of each nucleotide, 17.55 μL deionized water, 1 μL template DNA, 1 unit Taq-polymerase (Evrogen, Moscow). PCR performed at 94 °С (3 min), followed by 30 cycles at 94 °С (15 s), 50 °С (45 s), 72 °С (60 s) and a final one at 72 °С (8 min). PCR products were visualized on 1% agarose gels and later purified by ethanol and ammonium acetate (3 M). Both strands were sequenced on an Applied Biosystems 3500 DNA sequencer (Thermo Scientific, USA) following the manufacturer’s instructions.

For alignment of *COI* nucleotide sequences we used MUSCLE algorythm in the MEGA6 software (Tamura et al. 2013). The MEGA6 was used to calculate pairwise genetic distances Kimura 2-parameter (K2P) with codon position preferences: 1^st^, 2^nd^, 3^rd^ and noncoding sites (Kimura 1980). The Bayesian analysis was performed using MrBayes v.3.2.6 software (Ronquist and Huelsenbeck 2003; Ronquist et al. 2012) with settings suggested by Karmokov (2019), for 1 000 000 iterations and 1000 iterations of burn-in, nst = 6 (GTP + I + G). The phylogenetic trees resulting in Bayesian inference analyses were visualized and edited using FigTree v.1.4.3 software (http://tree.bio.ed.ac.uk/software/figtree/).

In addition, the forty one COI sequences of the genus *Chironomus* from “GenBank” and “Barcode of Life Data Systems” (BOLD)* were used for comparison. Accession numbers of used sequences in GenBank and BOLD: *Chironomusacutiventris* Wülker, Ryser et Scholl 1983 (AF192200.1), *Ch.annularius* Meigen, 1818 (AF192189.1), *Ch.aprilinus* Meigen, 1830 (KC250746.1), *Ch.balatonicus* Devai, Wulker et Scholl, 1983 (JN016826.1), *Ch.bernensis* Wülker et Klötzli,, 1973 (AF192188.1), *Ch.borokensis* Kerkis, Filippova, Schobanov, Gunderina et Kiknadze, 1988 (AB740261), *Ch.cingulatus* Meigen, 1830 (AF192191.1), *Ch.commutatus* Keyl, 1960 (AF192187.1), *Ch.curabilis* Belyanina, Sigareva et Loginova, 1990 (JN016810.1), *Ch.dilutus* Shobanov, Kiknadze et Butler, 1999 (KF278335.1), *Ch.entis* Shobanov, 1989 (KM571024.1), *Ch.heterodentatus* Konstantinov, 1956 (AF192199.1), *Ch.heteropilicornis* Wülker, 1996 (MK795770.1, MK795771.1, MK795772.1, CHMNO268-15*, CHMNO413-15, CHMNO267-15, CHMNO269-15, CHMNO266-15), *Ch.luridus* Strenzke, 1959 (AF192203.1), *Ch.maturus* Johannsen, 1908 (DQ648204.1), *Ch.melanescens* Keyl, 1961 (MG145351.1), *Ch.nipponensis* Tokunaga, 1940 (LC096172.1), *Ch.novosibiricus* Kiknadze, Siirin et Kerkis, 1993 (AF192197.1), *Ch.nuditarsis* Keyl, 1961 (KY225345.1), *Ch.obtusidens* Goetghebuer, 1921 (CHMNO207-15*); *Ch.piger* Strenzke, 1959 (AF192202.1), *Ch.pilicornis* Fabricius, 1787 (BSCHI736-17, BSCHI735-17, HM860166.1, ARCHR033-11, INNV033-08, ARCHR026-11, KR593529.1), *Ch.plumosus* Linnaeus, 1758 (KF278217.1), *Ch.riparius* Meigen, 1804 (KR756187.1), *Ch.tentans* Fabricius, 1805 (AF110157.1), *Ch.tuvanicus* Kiknadze, Siirin et Wülker, 1993 (AF192196.1), *Ch.whitseli* Sublette et Sublette, 1974 (KR683438.1). The *COI* gene sequence of *Ptychopteraminuta* Tonnoir, 1919 (KF297888) was used as outgroup in phylogenetic analysis.

## ﻿Results and discussion

The morphological characteristics of mentum, antenna, mandible and ventromental plate of the larva are presented in Fig. 2. In general the morphological characteristics are similar to those previously described in Kiknadze et al. (1996).

The head capsule is dark yellow. The mentum is black-brown with sharp teeth. The central tooth with small additional teeth (Fig. 2a). The third to fifth teeth are almost the same size and lighter in color than the first and second teeth. The sixth tooth a small and light. Basal segment of antenna (Fig. 2d) is cone-shaped, length 119–167 µm. Antenna blade is extended to the base of a fourth segment (Kiknadze et al. 1996), but on the fig. 6 (Kiknadze et al. 1996) it is extended to the middle of a fifth segment and similar to Fig. 2d. Ventromental plates (Fig. 2b) with small outer hooks, the number of striae is 64–84 (Kiknadze et al. 1996). Mandible (Fig. 2c) with black first and brownish second teeth. Three lower teeth are black. The fourth tooth is small, it is color varied from light to dark brown.

### ﻿Karyotype of *Chironomusheteropilicornis* Wülker, 1996 from the Gydan Peninsula

The chromosome set of the species is 2n = 8. The chromosome arm combination is AB, CD, EF and G (the *Chironomus* “*thummi*” cytocomlex). The additional B-chromosomes are absent. The chromosomes AB and CD are metacentric, EF is submetacentric, and G is telocentric. Nucleoli were found in arms B, D, E and G, Balbiani rings in arms B and G. The homologues in arm G usually laying closely to each other or are tightly paired (Kiknadze et al. 2016).

**Figure 3.**
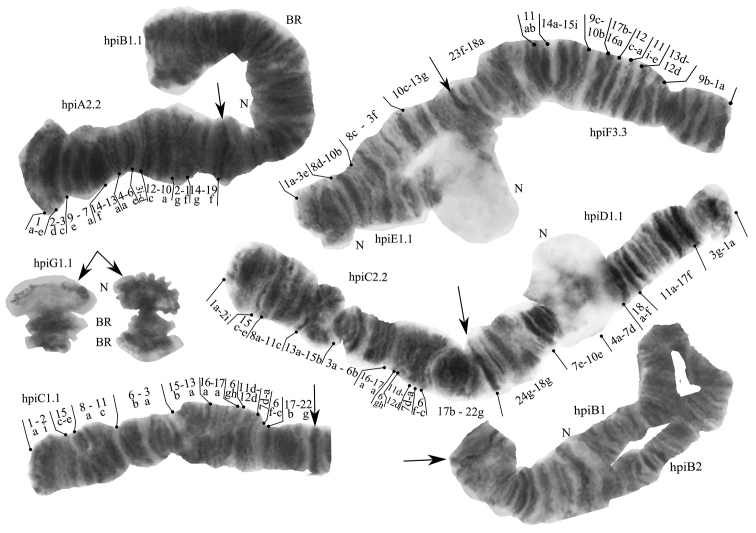
Karyotype of *Chironomusheteropilicornis* from the Gydan Peninsula, Russia. Arrows indicate centromeric band, hpiA2.2, hpiB1.1 and etc. – genotypic combinations of banding sequences in chromosome arms, BR – Balbiani rings, N – nucleous.

We found three different karyotypes in four larvae from the Gydan Peninsula: hpiA2.2.B1.1.C.1.1.D1.1.E.1.1.F.3.3.G1.1. (in two larvae), hpiA2.2.B1.2.C1.1.D.1.1.E.1.1.F.3.3. G1.1. and hpiA2.2.B1.1.C2.2.D.1.1.E.1.1.F.3.3.G1.1. They consist of 9 banding sequences out of 16 known for the banding sequences pool of this species (Kiknadze et al. 2016; Karmokov 2019) and one new hpiB2 sequence reported for the first time (Fig. 3). Sequences hpiA2 and hpiE1 mapped according to Karmokov (2019).

**Arm A.** One banding sequence hpiA2 1a-e 2d-3c 9e-7a 14f-13a 4a-6e 3i-d 12c-10a 2g-1f 14g-19f C.

**Arm B.** Two banding sequences: hpiB1was found in homozygous and heterozygous state with hpiB2, which was described for the first time. Frequency of sequences hpiB1 – 0.875 and hpiB2 – 0.125. Both banding sequences are still not mapped.

**Arm C.** Two banding sequences: hpiC1 1a-2i 15c-e 8a-11c 6b-3a 15b-13a 16a-17a 6gh 11d-12d 7d-a 6f-c17b-22g C and hpiC2 1a-2i 15c-e 8a-11c 13a-15b 3a-6b 16a-17a 6hg 11d-12d 7d-a 6f-c 17b-22g C. Frequency of sequences hpiC1 – 0.750 and hpiC2 – 0.250. Both sequences founded in homozygous state.

**Arm D.** One banding sequence: hpiD1 1a-3g 17f-11a 18f-a 7d-4a 10e-7e 18g-24g C.

**Arm E.** One banding sequence: hpiE1 1a-3e 8d-10b 10c-13g C.

**Arm F.** One banding sequence: hpiF3 1a-9b 12d-13d 11e-i 12a-c 16a-17d 10d-9c 15i-14a 11b-a 18a-23f C.

**Arm G.** One banding sequence: hpiG1 was found. Not mapped.

In total, nine banding sequences were found. The main feature of the population is the presence of rare banding sequences hpiA2 and hpiF3 only in the homozygous state. Another interesting moment is the large nucleous in D (7e-10e) and E (10c-11a) arms, usually, it is not so big. By the morphology, the chromosomes are similar to the karyotype of *Ch.heteropilicornis* from Netherlands (fig. 2.27.2, Kiknadze et al. 2016). Probably, it is a result of some non-obvious similar characteristics of water bodies, for example, a temperature. As we know, the characteristics of the karyotype and distribution of inversion variants in *Chironomus* depends more on the conditions in the local water body than on their geographic location (Gunderina et al. 1999), and the physiological condition of the organism (Iliinskaya 1984; Dyomin and Iliinskaya 1988; Dyomin 1989).

### ﻿DNA-barcoding and phylogenetic analysis

Eight sequences for *Ch.heteropilicornis* and seven for *Ch.pilicornis* were found in genetic information databases, GenBank and BOLD (see access numbers in material and methods), there are populations from Canada, Greenland, Sweden, Norway, and Georgia. We obtained the *COI* sequence barcode for *Ch.heteropilicornis* with the length of 617 nucleotides (percentage A: 25; T: 36; G: 18; C: 21) and deposited it into the GenBank database with accession number – MZ450155. The pairwise genetic distances between the members of the *Ch.pilicornis* group obtained by K2P model (Kimura 1980) shown high variability. Distance between sequences of *Ch.heteropilicornis* from the Gydan Peninsula and: Georgia was 1.8%, Norway – 1.1–1.3%, with *Ch.pilicornis* from Sweden – 1.1%, Canada and Greenland – 5.3%. According to Proulx et al. (2013)*Chironomus COI* interspecific sequence distances are about 3%. In our study, the distances between different populations of *Ch.heteropilicornis* varies from 1.1 to 1.8%, that is much lower than the 3% accepted interspecific threshold.

**Figure 4.**
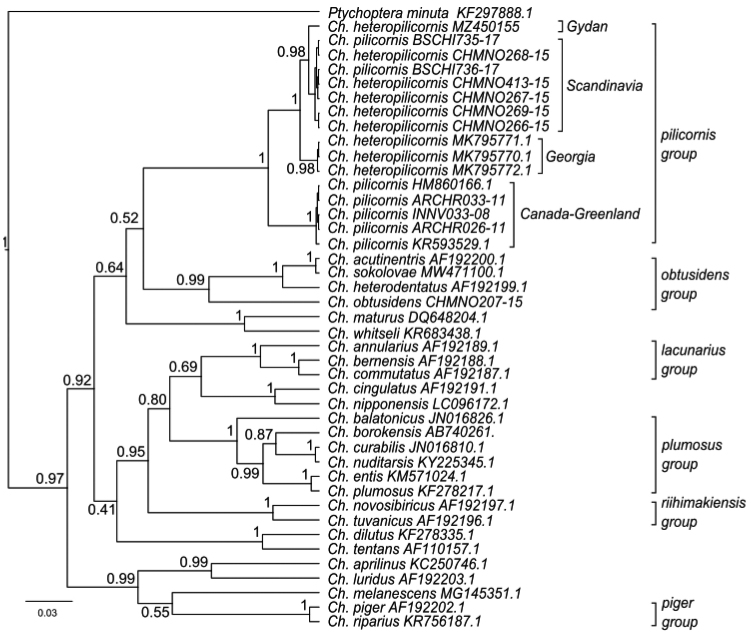
Bayesian tree of the analyzed samples of *Chironomus* spp. inferred from *COI* sequences. Species name, GenBank accession numbers and group name are shown to the right of the branches. Support values are given if they exceed 0.4. The numbers at the nodes indicate posterior probabilities.

The analysis of the phylogenetic tree constructed by Bayesian inference showed groups of sibling species (Fig. 4), and the *Ch.pilicornis* group is divided into geographically determined clusters: 1) Canada and Greenland, 2) Georgia, and Scandinavia (Norway, Sweden) and Gydan, with support value 0.98. Another interesting moment is the presence of two *Ch.pilicornis* sequences (BSCHI735-17, BSCHI736-17) along with the *Ch.heteropilicornis* sequences inside the Scandinavian cluster. If this is not a result of species misidentification, it could be a result of interspecific hybridization and horizontal transfer of mitochondrial genes with fixation in one of the parental species in the population (Guryev and Blinov 2002; Polukonova 2009; Polukonova and Dyomin 2010, 2013; Karmokov 2019; Bolshakov and Prokin 2021). About possibilities of hybridization between sibling-species in *Chironomus* are well known: *Camptochironomustentans* × *C.pallidivittatus* (Tichy 1975), *Ch.plumosus* × *Ch.muratensis* Ryser, Scholl et Wülker 1983, *Ch.muratensis* × *Ch.nudiventris* Ryser, Scholl et Wülker 1983, *Ch.plumosus* × *Ch.borokensis* (Butler et al. 1999), *Ch.riparius* × *Ch.piger* (Petrova et al. 2014). Karmokov (2019) suppose that interspecific hybridization event between *Ch.heteropilicornis* (female) and *Ch.pilicornis* (male) in the population of Sweden, because according to Wülker (1996) both species occurred sympatrically in collection site Kyrkösjärvi, Seinajöki-area (South Ostrobothnia, western Finland) which not so far from the place where were collected specimens of *C.pilicornis* (BSCHI735-17, BSCHI736-17) from BOLD.

## ﻿Conclusions

*Chironomusheteropilicornis* is recorded from the Gydan Peninsula for the first time. Three different karyotypes in four larvae were found. The hpiB2 banding sequence is new for the species. The karyotypes of the population have a characteristic feature, possession of hpiA2 only in a homozygous state and phiF3 has been observed only in the homozygous state for the first time, and unusually large nucleous in D and E arms. We found sequences hpiA2.2, hpiC1.1, hpiD1.1 and hpiE1.1 in all larvae. The same situation with the occurrence of these banding sequences was in all of 33 Georgian individuals (Karmokov 2019). The sequence hpiF3 was found in all larvae from the Gydan Peninsula, absent in Georgia (Karmokov 2019), but present in Yakutian populations with an occurrence from 9 to 22.5% (Kiknadze et al. 1996).

On the phylogenetic tree constructed by the Bayesian inference, we can see clusters of the sibling species groups: *Ch.obtusidens*, *Ch.lacunarius*, *Ch.plumosus*, *Ch.riihimakiensis*, *Ch.piger* and *Ch.pilicornis*, that were independently identified based on morphological and cytogenetic characteristics. In the *Ch.pilicornis* group, we can see the clusters explained geographically: Canada-Greenland and Georgia-Scandinavia-Gydan. The geographic distance in latitudes between Gydan and Georgian populations is about 3000 km, with Scandinavian populations 400–800 km and 400–1000 km with Greenland and Canada. We can conclude that the conditions in closely located sites will be similar, for example, in the Tundra zone it is the predominance of negative air temperatures per year, a predominance of oligotrophic waters, etc.

Unfortunately, we have no opportunity to examine the karyotype of the *Ch.pilicornis* from Canada. The genetic distances between most of the Palearctic and Canadian populations are 5.1%, as well as Greenland one (Karmokov 2019), that is more than the 3% accepted interspecific threshold (Proulx et al. 2013). A similar situation is known in the *Camptochironomus* group, for karyotypes and morphological characteristics of *C.tentans* and *C.dilutus*, which diverged during a long period of continental isolation to independent species (Shobanov et al. 1999; Kiknadze et al. 2007). Thus, the Canada-Greenland cluster is characterized by long isolation from other populations and can, possibly, represent one new, separate species.

Four larvae are not enough for complete chromosomal polymorphism analysis. Based on all the available data on karyotype and *COI* gene sequences, we can conclude that the population of *Ch.heteropilicornis* from the Gydan Peninsula has an intermediate position between Georgian (hpiA2.2), Yakutia (hpiF3.3) and Scandinavian (*COI*) populations within the European cluster. The absence of Yakutian population DNA-sequencing and data from other Asian regions gives no chance to establish a phylogeographical scenario for *Ch.heteropilicornis* at the moment.

## Supplementary materials

Supplementary material 1 Fig. 6 from Kikanadze et al. 1996 Data type pdf file Explanation note Karyotypes of kryolitozone of Yakutya. https://binary.pensoft.net/file/619613This dataset is made available under the Open Database License (http://opendatacommons.org/licenses/odbl/1.0/). The Open Database License (ODbL) is a license agreement intended to allow users to freely share, modify, and use this Dataset while maintaining this same freedom for others, provided that the original source and author(s) are credited.Kikanadze et al.
