# Supplementary figures and images for: ﻿Karyotype and COI gene sequence of Chironomusheteropilicornis Wülker, 1996 (Diptera, Chironomidae) from the Gydan Peninsula, Russia
Source: Comp Cytogenet. 2021 Dec 7;15(4):447–58. doi: 10.3897/CompCytogen.v15i4.73135 (PMC8671704; doi:10.3897/CompCytogen.v15i4.73135)

Кикнадзе и др., 1996. Кариотипы криолитозоны Якутии.

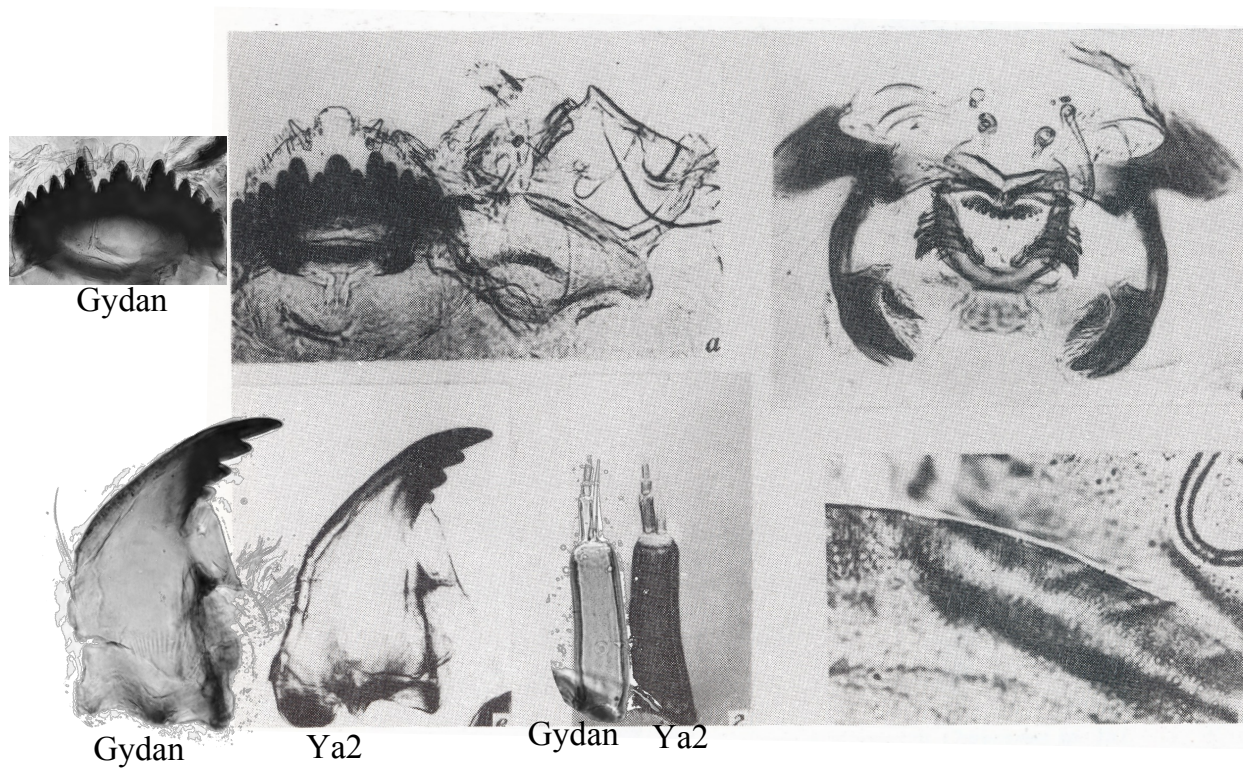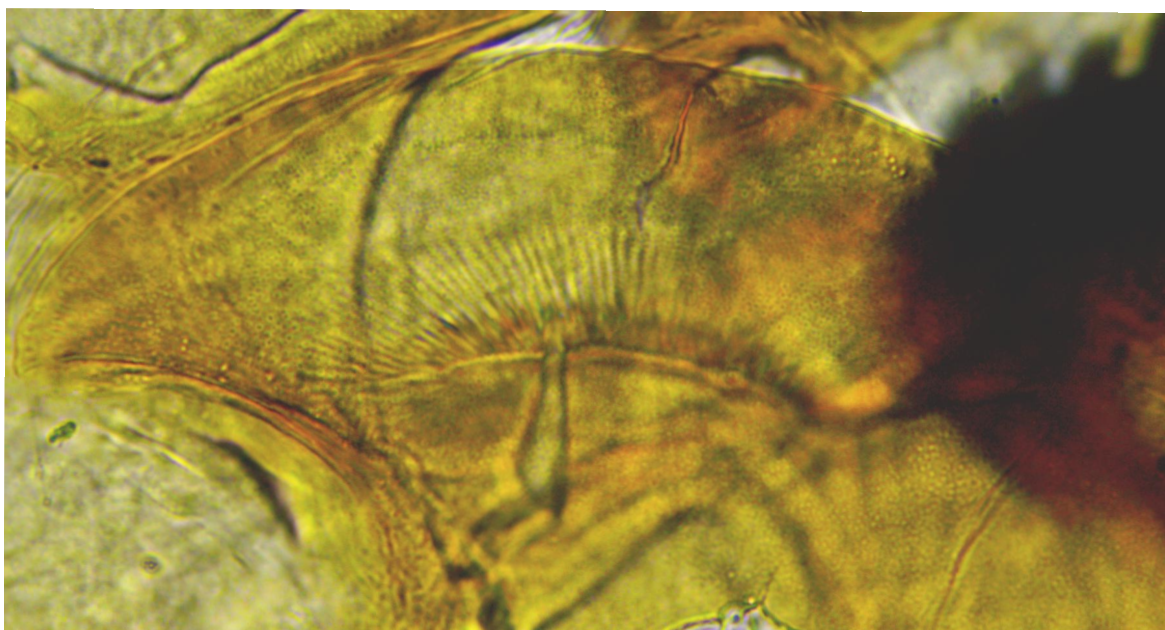

Supplement: Supplementary material 1 — Fig. 6 from Kikanadze et al. 1996 [file comparative_cytogenetics-15-447-s001.pdf]
